# Extradigital Glomus Tumor Mimics an Intrinsic Nerve Tumor in a Trauma Patient: Case Report and Literature Review

**DOI:** 10.7759/cureus.19256

**Published:** 2021-11-04

**Authors:** Rodiyah T Ajala, Kristopher A Lyon, Priscilla R Lyon, Frank S Harris

**Affiliations:** 1 Surgery, Texas A&M University College of Medicine, Bryan, USA; 2 Neurosurgery, Baylor Scott & White Medical Center – Temple, Temple, USA; 3 Pathology, Baylor Scott & White Medical Center – Temple, Temple, USA

**Keywords:** traumatic neuroma, trauma, peripheral neuropathy, nerve tumor, extradigital glomus tumor

## Abstract

Glomus tumors are rare, painful, and usually benign neoplasms that typically occur at the subungual aspect of digits. Rarely, glomus tumors may arise in other areas of the body. We present a case of an extradigital glomus tumor on a forearm with prior trauma that presented with symptoms of an isolated peripheral neuropathy. Our review of literature reveals how upper or lower extremity glomus tumors can mimic neuropathies secondary to intrinsic nerve tumors (schwannoma, neurofibroma, or neuroma), radiculopathies, or manifestations of a complex regional pain syndrome (CRPS). We emphasize the need to consider a broad differential diagnosis that includes glomus tumor when evaluating patients with painful dermal masses producing peripheral neuropathy or radiculopathy signs owing to the infiltrative growth pattern into or mass effect exerted on nearby nerves.

## Introduction

Glomus tumors are very painful neoplasms arising from glomus cells of glomus bodies, which are specialized arteriovenous structures within the subcutaneous connective tissue of skin involved in body temperature regulation by regulating blood flow [1,2]. These glomus cells are modified smooth muscle cells with contractile abilities and they surround the arterial end of a glomus body, which is also known as Sucquet-Hoyer canal [3,4]. Glomus tumors occur mostly as a solitary mass at the subungual area of digits because these areas are rich in glomus bodies [1]. Extradigital glomus tumors are found in different parts of the upper and lower extremities as well as the nose, cheek, ear lobe, bone, back, stomach, lungs, trachea, and fallopian tube [1]. Because of this, glomus tumors can present with varying non-specific localizing signs that may lead to misdiagnosis. Glomus tumors classically present with a triad of symptoms - pain, focal tenderness, and cold hypersensitivity. However, cold intolerance is often absent as a reported symptom of extradigital glomus tumors of upper or lower extremities. Complete surgical excision is curative, so it is crucial that physicians have a high level of suspicion and include glomus tumor in the differential diagnosis when patients present with an isolated peripheral neuropathy or radiculopathy symptoms in the setting of an unrevealing spine survey [3,5,6].

## Case presentation

A 48-year-old Caucasian man with past medical history of asthma, diverticulitis, benign prostatic hyperplasia, presbyopia, and obesity presented with a tender palpable left forearm mass. The pain started five years after the patient sustained a deep knife wound in this area that required stitches. Initially, without significant discomfort, the mass progressively grew for years. The pain worsened significantly in the last eight months prior to presentation as he began experiencing dysesthesias that radiated distally to his thumb, index, and middle fingers when the mass was lightly touched. The pain had become so bothersome to the patient that even the wind blowing across his forearm caused extreme discomfort. It affected his livelihood as a truck driver and was deterring him from playing golf. 

**Figure 1:**
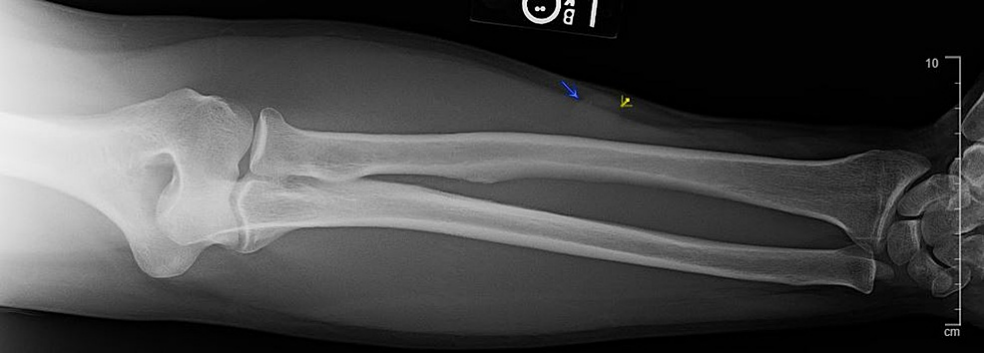
Plain radiograph image of left forearm shows 1.6 cm ovoid density within the lateral soft tissues.

**Figure 2:**
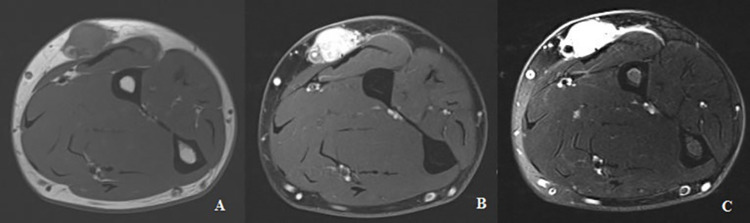
MRI of left forearm, axial views. A: T1-weighted image without contrast that shows low-intensity well-circumscribed mass; B: T1-weighted image with contrast that shows strong enhancement of mass; C: T2-weighted image that shows hyperintense mass.

**Figure 3:**
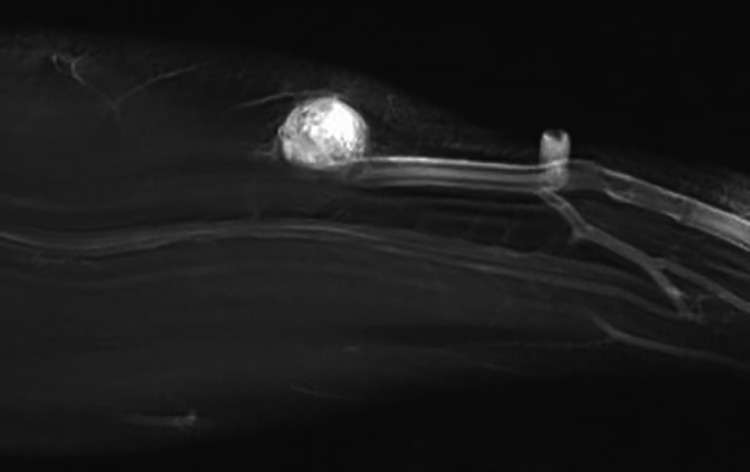
MRI of left forearm, sagittal view. T1-weighted image with contrast shows contrast-enhancing mass compressing the superficial branch of the radial nerve and associated vascular bundle.

Plain radiograph revealed a 1.6 cm ovoid density within the lateral soft tissues of the left forearm (Figure 1). Subsequently, a MRI of the left forearm was taken, which showed a 1.6 x 1.3 x 1.8 cm subcutaneous mass with low T1-weighted signal intensity that avidly enhanced with contrast and high-intensity signal on T2-weighted imaging (Figure 2). The mass was closely associated with the neurovascular bundle following the course of the superficial branch of the radial nerve (Figure 3). This led to the initial conclusion of the mass resembling an intrinsic nerve tumor such as schwannoma, neurofibroma, or a traumatic neuroma. 

On physical examination, light touch of the mass elicited significant discomfort and dysesthesias in the distribution of superficial branch of radial nerve, but sensation to both light touch and pinprick was intact as well as the motor function of the entire hand. No atrophy of the hand muscles was observed. Considering the patient’s prior trauma to the forearm at the same location as the tumor and the imaging features, the presumptive diagnosis became neuroma, a reactive neoplasm that can arise from an injured nerve [7]. 

The patient was taken to the operating room where general anesthesia was administered. The left forearm was prepped and draped in standard fashion. A 5 cm curvilinear incision was made over the palpable mass in the left forearm. The mass was seen just below the subcutaneous fatty tissue superficial to the fascia. A small nerve fiber was seen in the vicinity of the mass, appearing to be contiguous with the mass. Vessel loops were used to isolate the nerve fiber, and the nerve was directly stimulated. There was no movement in the hand with direct intraoperative stimulation. The mass was slowly dissected in a proximal to distal fashion off the nerve. The mass appeared to have a robust blood supply that was controlled with bipolar coagulation. The mass was grossly resected, and hemostasis was achieved. At the two-week follow-up, the patient reported significant improvement in his pain, mentioned mild tingling around the site of incision, and resolution of his pre-operative pain symptoms. 

**Figure 4:**
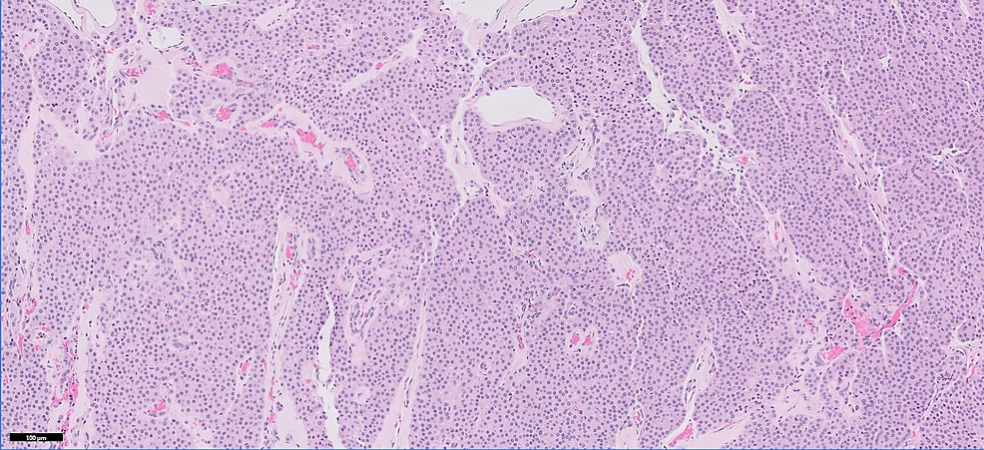
Dense aggregates of glomus cells with characteristic round cells with bland round nuclei and interspersed small hyalinized vessels (H&E, 100x). H&E: hematoxylin and eosin

**Figure 5:**
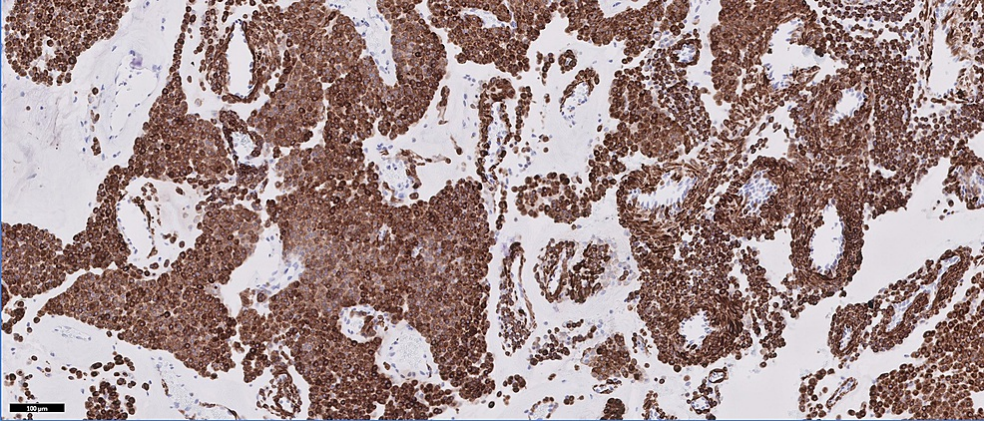
Constituent cells are diffusely positive for calponin (IHC, 100x). IHC: immunohistochemistry

Histologic examination demonstrated a well-circumscribed, encapsulated soft tissue nodule consisting of sheets, nests, and trabeculae of glomus cells interspersed with small hyalinized vessels (Figure 4). Immunohistochemical stains showed the glomus cells were positive for calponin (Figure 5) and smooth muscle actin, and negative for S100, chromogranin, synaptophysin, epithelial membrane antigen, and desmin. CD34 highlighted the vessels. The diagnosis of glomus tumor was rendered.

## Discussion

Glomus tumors account for one to six percent of all diagnosed soft tissue tumors, and there is significant heterogeneity in the clinical presentation of these tumors [1,4]. In a retrospective study performed by Schiefer et al. over a 20-year period, only about two percent of patients with extradigital glomus tumor presented with the classic symptom triad of pain, pinpoint tenderness, and cold hypersensitivity; most patients did not present with cold hypersensitivity [1]. Another retrospective comparative study by Lee et al. suggested that the high expression levels of substance powder (P) and transient receptor potential cation channel subfamily V member 1 (TRPV1) in classical and extradigital glomus tumors implicate them possibly as molecular mediators for the excruciating pain and cold hypersensitivity experienced by patients [8]. Nevertheless, the cold hypersensitivity is often missed objectively during a physical examination of an extradigital glomus tumor resulting in misdiagnosis or delayed diagnosis [4,8]. This translates to prolonged duration of chronic excruciating pain or psychiatric diagnosis for patients in many cases especially if the tumor size is so small that it can’t be easily palpated [1]. The pain described by patients is often disparate from the observed tumor size [3,9]. For this case presentation, the clinical suspicion was peripheral neuropathy from a traumatic neuroma based on the patient’s history of a deep laceration in the exact location as the tumor, the MRI findings, and the radiating dysesthesias to the first three digits. However, the histopathological findings showed that the tumor was not of neural origin since immunohistochemical staining was negative for synaptophysin, chromogranin, and S-100. 

Trauma in association with the formation of a glomus tumor has been mentioned in the literature, but the possible underlying pathogenesis has not been elucidated. Of patients studied by Schiefer et al., 20% reported trauma to the site of the extradigital glomus tumor before the onset of symptoms [1]. Lunn et al. reported a misdiagnosis of postoperative neuroma following an elbow surgery that turned out to be a glomus tumor after histopathologic analysis [6]. The presenting symptoms and differential diagnosis considered for cases of extradigital glomus tumors arising in upper or lower extremities in association with prior trauma are seen in Table 1 [3,6,10-14]. Trauma is a common etiology for the presentation of radiating pain or dysesthesia, hyperesthesia, paresthesia, or allodynia as manifestations of neurological disorders such as peripheral neuropathy, intrinsic nerve tumors (schwannoma, neurofibroma, or neuroma), radiculopathy caused by intra- or extra-spinal mass lesions, and complex regional pain syndrome (CRPS) [7,15]. They can have other accompanying sensory or motor deficits such as numbness or ataxia, which were absent in this case. To differentiate between these conditions, a careful history, physical examination, and further investigations with imaging such as MRI, CT, or USG are usually performed. 

**Table 1:** Extradigital glomus tumors of the upper and lower extremities associated with a history of local trauma. CRPS: complex regional pain syndrome; F: female; IHC: immunohistochemistry; L: left; M: male; R: right; ROM: range of motion; SMA: smooth muscle actin

Author, Year	Age, Sex	Tumor Site	Presentation	Palpable Tumor/Mass	Tumor Dimensions	Differential Diagnosis	Mode of Diagnosis	Prior Trauma	Outcome and Follow-Up
Chun et al., 2013 [3]	45 years, M	Elbow	Pain for two weeks	Yes	0.9 x 0.7 cm	Hemangioma	Histopathology with IHC	Puncture wound on a tree two weeks prior	Unknown
Lunn et al., 2016 [6]	47 years, M	L elbow, medial epicondyle	Severe localized pain for eight months	Yes	14 x 6 x 10 mm	Medial epicondylitis	Histopathology with IHC positive for SMA	Prior surgery	Asymptomatic after surgical excision and at 15-months follow-up
Nigam et al., 2012 [10]	45 years, M	L forearm, flexor aspect	Pain for five years	Yes	0.5 x 0.5 cm	Hemangioma, glomus tumor, eccrine spiradenoma	Histopathology with IHC	Previous excisional surgery	Unknown
Chim et al., 2017 [11]	47 years, M	R antecubital fossa	Debilitating pain, hypersensitivity, pain with flexion	Unknown	2 mm diameter	Neuroma of lateral antebrachial cutaneous nerve	Histopathology	Repair of distal biceps tendon rupture and neurolysis of lateral antebrachial cutaneous nerve on separate occasions	Asymptomatic after surgical excision, unknown follow-up duration
Christian, 2020 [12]	67 years, M	L knee	Pain, hyperesthesia for 21 years, noticed ‘bump’ for several months	Yes	10 x 15 x 5 mm	Glomus tumor	Clinical findings, confirmed with histopathology and IHC positive for SMA	Ruptured quadriceps tendon repair 21 years prior	Asymptomatic after surgical excision and at three years follow-up
Wong et al., 2015 [13]	51 years, M	R knee, medial aspect of quadriceps tendon insertion	Pain, exquisite tenderness for 10 years, altered gait, decreased ROM	No	7 mm	Medial plica syndrome, quadriceps tendonitis, chondromalacia patella, saphenous neuritis, CRPS, ganglion cyst, peripheral nerve sheath tumor	Histopathology with IHC positive for caldesmon and SMA	Low level trauma	Asymptomatic after surgical excision, unknown follow-up duration
Friske et al., 2016 [14]	33 years, M	R wrist, ulnar aspect	Pain, localized tenderness for two years, worsened in last three to four months	Yes	2.0 x 1.6 x 1.5 cm	Sarcoma, chondroma, soft tissue hemangioma, foreign body granuloma	Histopathology with IHC positive for SMA, vimentin, desmin, and CD34	Injury with glass foreign debris two years prior	Unknown
Present case, 2021	48 years, M	L forearm	Pain, localized tenderness, dysesthesias for 33 years worsened in last eight years	Yes	1.6 x 1.3 x 1.8 cm	Schwannoma, traumatic neuroma, neurofibroma	Histopathology with IHC positive for calponin, SMA, CD34	Deep knife wound	Asymptomatic after surgical excision and at two weeks follow-up

Owing to the benign nature of most glomus tumors, they grow slowly, and the symptom onset is typically insidious like in this case. This slow growth potentially translates to the mass effect that a glomus tumor can have to present like a progressive peripheral neuropathy. In our case, the patient experienced pain that progressively worsened in the forearm, which likely indicated a mass effect of the glomus tumor on the lateral antebrachial cutaneous nerve in addition to the superficial branch of the radial nerve. Similarly, Smith reported an unusual case of a patient demanding an arm amputation after his extradigital glomus tumor at the metacarpal base of his small finger was misdiagnosed as CRPS secondary to ulnar neuropathy for over nine years [16]. Schiefer et al. also found that only nine percent of the 56 patients they analyzed had glomus tumors considered as part of their initial evaluation. Most often, the misdiagnoses were hemangioma, neuroma, or neurofibroma [1]. 

Only histopathological analysis often aided by immunohistochemistry is used to make a definite diagnosis of glomus tumor although a high clinical suspicion is necessary for it to be considered in the differential diagnosis [7,9]. It is frequently mentioned in the literature that MRI is the imaging modality of choice for the diagnosis of glomus tumors. However, in this case and other case reports, it misdiagnosed the tumor but localized the tumor size and anatomy for curative surgical excision [9,11,12]. Color Doppler USG is another imaging modality often reported in the literature that can aid in the diagnosis and pre-operative localization of glomus tumors [12,15,17]. It can visualize the arterial patterns as hypervascularity of the mass with color Doppler, which can be used to distinguish glomus tumors from other soft tissue tumors. Glomus tumors present as well-circumscribed, hypervascularized, hypoechoic subcutaneous solid tumors on USG [15,17]. Additionally, USG doesn’t require intravenous administration of contrast agents or radiation [15]. It is important to note that hypervascularity may not be reliable for detecting all glomus tumors since only two out of the three known histologic variants are highly vascular - glomangioma type with prominent vasculature, and glomangiomyoma type with prominent vasculature and smooth muscle [9,13]. The last histologic variant is solid glomus tumor type with poor vasculature and smooth muscle. 

The reported reoccurrence rates of benign glomus tumors are in the range of 12-33% and are often due to incomplete resection, so careful and complete excision of the glomus tumor is essential [1,3,14]. Malignant glomus tumors are extremely rare but, they typically have very high mitotic activity, are greater than 2 cm in size, and arise in deep locations [1,3].

## Conclusions

Extradigital glomus tumors are typically benign, soft tissue tumors that presents as a dermal nodule with one or more symptoms from the classic triad of pain, focal tenderness, and cold insensitivity. The nodule may be palpable and the typical MRI findings of low signal intensity on T1-weighted imaging, higher intensity signals with contrast, and T2-weighted imaging should raise the suspicion of a glomus tumor. Color Doppler USG can be used to differentiate glomus tumor variants with prominent vasculature from other soft tissue tumors. Ultimately, to confirm the diagnosis, surgical resection with subsequent histopathologic analysis is required.

A history of prior trauma may be the cause for extradigital glomus tumors to arise. The pathophysiology of glomus tumors secondary to trauma requires further investigation. Since the presenting signs and symptoms of an extradigital glomus tumor mimic those of peripheral neuropathy, providers need to consider a broad differential diagnosis when evaluating patients in the setting of an unrevealing spine survey. Patients with painful, superficial dermal masses or non-palpable masses seen only on imaging adjacent to peripheral nerves may harbor a glomus tumor instead of an intrinsic nerve tumor.
